# Tyrosinase Inhibitory Activity of Soybeans Fermented with *Bacillus subtilis* Capable of Producing a Phenolic Glycoside, Arbutin

**DOI:** 10.3390/antiox9121301

**Published:** 2020-12-18

**Authors:** Young Hun Jin, Ah Ran Jeon, Jae-Hyung Mah

**Affiliations:** Department of Food and Biotechnology, Korea University, 2511 Sejong-ro, Sejong 30019, Korea; younghoonjin3090@korea.ac.kr (Y.H.J.); j_ahran@korea.ac.kr (A.R.J.)

**Keywords:** fermented soybean, tyrosinase inhibitory activity, skin lightening, anti-neurodegenerative activity, arbutin production, *Bacillus subtilis*, *Cheonggukjang* model

## Abstract

The production of arbutin, an effective tyrosinase inhibitor as well as an outstanding antioxidant, by 691 *Bacillus* strains isolated from soybean-based foods was tested to enhance the tyrosinase inhibitory activity of soybeans via fermentation with the strains. Among the strains tested, the 5 strains capable of significantly producing arbutin were identified as *B. subtilis* via 16S rRNA sequencing. When soybeans were fermented with each of the selected strains, the arbutin content was highest on day 1 of fermentation and decreased thereafter. However, the tyrosinase inhibitory activity of the fermented soybeans continuously increased as fermentation progressed, whereas the activity of non-inoculated soybeans was consistently low. The results indicate that arbutin enhances the tyrosinase inhibitory activity of soybeans in the early period of fermentation, while other substances besides arbutin contribute to the activity in the later period. Consequently, soybeans fermented with arbutin-producing *B. subtilis* strains could be considered as a natural source of cosmeceuticals and nutricosmetics used in skin lightening and may be of interest in the food industry because they contain well-known and powerful antioxidants such as arbutin and other substances.

## 1. Introduction

Melanin plays an important role in preventing skin damage induced by ultraviolet (UV) irradiation [1]. The synthesis of melanin is mainly regulated by tyrosinase [2]. L-tyrosine, a precursor of melanin, is converted by tyrosinase into L-3,4-dihydroxyphenylalanine (L-DOPA), spontaneously oxidized, and subsequently polymerized to form melanin [2]. However, long-term exposure to UV irradiation can lead to abnormally increased melanin synthesis (hyperpigmentation) by tyrosinase [1], and the hyperpigmentation is one of the major stimulants for skin diseases such as freckles, senile lentigines, and even melanoma [3]. Consequently, melanin has been labelled as a “two-edged sword”; both protecting the skin against UV irradiation and oxidative stress and holding the risk of inducing skin diseases [4]. Meanwhile, tyrosinase is also involved in neuromelanin synthesis in the brain [5]. Like melanin, L-DOPA converted from L-tyrosine by tyrosinase is successively oxidized and polymerized to form neuromelanin [6]. The intraneuronal neuromelanin could play a protective role by preventing the accumulation of catechol derivatives and by scavenging reactive materials [7]. On the contrary, extraneuronal neuromelanin plays a toxic role in the aggravation of neurodegeneration by stimulating the release of neurotoxic molecules such as tumor-necrosis factor α, interleukin 6, and nitric oxide [7]. In recent studies [8,9], tyrosinase has been considered to be implicated in neurodegenerative disorders like Parkinson’s disease. In addition, the toxicity of dopamine (produced by tyrosinase) could be intensified by overexpression of the enzyme, mainly found in patients with Parkinson’s disease [10]. To prevent and/or treat such diseases, some compounds and natural extracts with tyrosinase inhibitory activity and antioxidative activity have been applied in the medicine and cosmetics industries [11,12], as well as the food industry [8].

Arbutin, kojic acid, azelaic acid, glycolic acid, resveratrol, and epigallocatechin gallate are major natural tyrosinase inhibitors which have been reported to produce a skin-lightening effect and/or anti-neurodegenerative activity by inhibiting the conversion of L-tyrosine into L-DOPA, and are known to be outstanding antioxidants [13,14,15,16]. Among them, arbutin, a phenolic glycoside, has been commonly applied to skin-lightening cosmetic products and suggested as a candidate for the treatment of Parkinson’s disease [13,17]. Numerous studies have suggested industrial production of arbutin through chemical synthesis [18], extraction from plants [12], and microbial enzymatic biotransformation [19]. Microorganism-mediated biotransformation has been considered an ideal method for arbutin production in the industries mentioned above, due to the manufacturing process under mild reaction conditions and the absence of toxic byproducts during the process [20]. *Bacillus subtilis*, *Leuconostoc mesenteroides*, *Xanthomonas campestris*, and other microorganisms have been employed for arbutin production [21]. Recently, several studies have reported that arbutin production can be enhanced by employing engineered microorganisms [22].

Fermented and non-fermented soybean foods have been abundantly consumed in Asian countries due to their extensive historical use and outstanding nutritional value [23]. Besides, the consumption of soybean foods is increasing worldwide due to the well-known beneficial functions of soybeans [23]. In 1999, the US Food and Drug Administration (FDA) approved the claim that soy protein may reduce the risk of coronary heart disease [24]. Soybean extract and other soybean-derived compounds such as isoflavones and peptides also have various health-promoting effects, including antioxidant [25], anti-inflammatory [26], anticancer [27], and tyrosinase inhibitory activity [28]. Furthermore, the health-promoting activities of soybeans can be enhanced by fermentation using microorganisms. For instance, the concentrations of daidzein, flavonoids, and other phenolic compounds steadily increased during the fermentation of *Cheonggukjang* (Korean traditional fermented whole soybean paste) inoculated with *B. pumilus* [29] or *B. subtilis* [30]. In another study, *B. coagulans* raised the content of daidzein, genistein, and glycitein during the fermentation of *Thua nao* (Thai traditional fermented soybean) [31]. Zhu, Fan, Cheng and Li [32] found that the antioxidant activity of *Meitauza* (Chinese traditional fermented okara) fermented with *B. subtilis* increased as fermentation progressed. Thus, fermented and non-fermented soybean foods have been regarded as potential sources of functional compounds as well as beneficial microorganisms [33].

Particularly, several studies have reported that the extracts of fermented soybean foods such as *Cheonggukjang* and *Doenjang* (Korean traditional fermented soybean paste) have strong tyrosinase inhibitory activity, as well as antioxidative activity [34,35,36]. Although the contribution of the fermenting microorganisms to antioxidative activity has been intensively studied [37,38], their effects on tyrosinase inhibitory activity in fermented soybean foods have been insufficiently reported. Therefore, this study was conducted to investigate the effect of arbutin-producing *B. subtilis* strains on the enhancement of tyrosinase inhibitory activity of fermented soybeans. For this purpose, arbutin-producing *B. subtilis* strains were isolated from representative soybean-based foods. Subsequently, the arbutin content and tyrosinase inhibitory activity of soybeans fermented with the strains were examined during the fermentation period. In addition, the contribution of arbutin to the total tyrosinase inhibitory activity of fermented soybeans was discussed.

## 2. Materials and Methods

### 2.1. Isolation and Identification of Bacillus Strains from Soybean-Based Products

Representative soybean-based products, including tofu, *Cheonggukjang* (Korean traditional fermented whole soybean paste), *Gochujang* (Korean traditional fermented red pepper paste), *Doubanjiang* (Chinese traditional fermented red pepper paste), *Chunjang* (Koreanized Chinese black soybean paste), *Natto* (Japanese traditional fermented whole soybean paste), and *Miso* (Japanese traditional fermented soybean paste), were purchased from several retail markets in Sejong, Korea. The products were immediately transported to the laboratory and stored at 4 °C until experimentation. Within 24 h of storage, *Bacillus* strains were isolated from the soybean products.

To isolate *Bacillus* strains, a 10 g sample of each product was homogenized with 90 mL of sterile 0.1% peptone saline using a stomacher (Laboratory Blender Stomacher 400, Seward, Ltd., Worthing, UK). The homogenate was 10-fold serially diluted with sterile 0.1% peptone saline. A 100 μL aliquot of each dilution was spread on plate count agar (PCA; Difco, Becton Dickinson, Sparks, MD, USA) in duplicate and incubated at 37 °C for 24 h. After incubation, all colonies on plates with 10–300 colonies [39] were streaked on PCA to isolate individual bacterial strains and incubated under the same conditions. The single colonies were streaked again on PCA and incubated under the same conditions to obtain pure cultures. Then, the single colonies were transferred into 5 mL of tryptic soy broth (TSB; Difco) and incubated under the same conditions. The cultured broth was stored in a deep freezer (-70 °C) using sterile glycerol (a final concentration of 20%, *v*/*v*).

*Bacillus* spp. were characterized and selected based on the morphological, cultural, and biochemical characteristics described in Bergey’s manual [40]. The *Bacillus* strains were further identified to the species level based on 16S rRNA gene sequence analyses. The universal bacterial primer pair 518F (5′-CCAGCAGCCGCGGTAATACG-3′) and 805R (5′-GACTACCAGGGTATCTAAT-3′) (all from Solgent Co. Daejeon, Korea) were used for the amplification of 16S rRNA gene. The identities of sequences were determined using the basic local alignment search tool (BLAST) of the National Center for Biotechnology Information (NCBI; http://www.ncbi.nlm.nih.gov/BLAST/).

*B. subtilis* KCTC 3135, *B. licheniformis* KCTC 1918, *B. coagulans* KCTC 3625, and *B. pumilus* KCTC 3855 were purchased from the Korean Collection for Type Cultures (KCTC; Daejeon, Korea) and served as reference strains to which the isolated strains were compared.

### 2.2. Preparation of Assay Medium for Arbutin Production and Arbutin Culture for HPLC Analysis

Arbutin production by *Bacillus* strains was determined using the procedure described by Liu et al. [41] with minor modifications. The assay medium for bacterial arbutin production was prepared with 20.00 g/L of sucrose, 10.00 g/L of peptone, 0.50 g/L of MgSO_4_, 1.00 g/L of K_2_HPO_4_, 1.00 g/L of KH_2_PO_4_, 2.00 g/L of NaCl, and 1.00 g/L of NaHCO_3_ (all from Sigma-Aldrich Chemical Co., St. Louis, MO, USA). The pH value of the assay medium was adjusted to 7.00 using 2.00 M NaOH (Sigma) solution, and the assay medium was autoclaved at 121 °C for 20 min.

A loopful (10 μL) of glycerol stock of each *Bacillus* strain (either each reference strain or each isolated strain selected based on arbutin production capability, refer to Section 3.1) was inoculated in 5 mL of TSB and incubated at 37 °C for 24 h. After incubation, 100 μL of the cultured broth was transferred into 5 mL of TSB and incubated under the same conditions. A loopful of the broth was streaked on tryptic soy agar (TSA; Difco). After incubation at 37 °C for 24 h, a single colony was inoculated in 5 mL of the bacterial arbutin production assay medium. After incubation at 37 °C for 15 h, hydroquinone (a precursor of arbutin) and sucrose (a donor for arbutin) dissolved in deionized water were filtered using a 0.45 μm-pore size sterile syringe filter (Millipore Co., Bedford, MA, USA) and then aseptically added into the cultured assay medium at final concentrations of 120 mM and 240 mM, respectively. The cultured assay medium with the aforementioned supplements was incubated again at 37 °C for 72 h, which is hereafter referred to as “arbutin culture”. The arbutin culture was immediately analyzed by HPLC for bacterial arbutin production.

### 2.3. Preparation of Bacterial Suspension for Soybean Fermentation

The bacterial suspension was prepared according to the procedure described in a previous study [42]. Briefly, 100 μL of the glycerol stock of each arbutin-producing *B. subtilis* strain (either reference strain or each isolated strain selected based on arbutin production capability, refer to Section 3.1) was inoculated in 5 mL of TSB and incubated at 37 °C for 24 h. After incubation, 100 μL of the cultured broth was transferred into 5 mL of TSB and incubated under the same conditions. To obtain a sufficient amount of bacterial cell culture, 5 mL of the culture were transferred into 250 mL of TSB. After incubation at 37 °C for 24 h, the cultured broth was centrifuged at 15,000× *g* for 5 min at 4 °C. The supernatant was discarded, and the pellet was washed three times and resuspended in a sterile M/15 Sörensen’s phosphate buffer. The buffer was prepared as follows: 5.675 g of Na_2_HPO_4_ and 3.630 g of KH_2_PO_4_ (all from Sigma) were dissolved in 1 L of distilled water and autoclaved at 121 °C for 15 min. The final concentration of bacterial cells in the suspension was adjusted to 8 log CFU/mL, and the bacterial suspension was further used for soybean fermentation (see Section 2.4).

### 2.4. Soybean Fermentation with Arbutin-Producing B. subtilis Using a Cheonggukjang Model

The soybean fermentation experiments were conducted following the protocol described in previous studies [43,44]. White soybeans (*Glycine max* Merrill) were purchased from a retail market in Sejong, Korea. The soybeans were washed in tap water three times and soaked in distilled water at 4 °C for 12 h. After draining for 5 min, 200 g of soaked soybeans were collected in a stainless container (190 × 160 × 50 mm^3^) and sealed with a stainless rubber-packing cover. The container was steamed at 121 °C for 40 min using an autoclave. After cooling to 50 °C, bacterial suspension was added to the soybeans at a final concentration of approximately 6 log CFU/g. The inoculated soybeans in the covered stainless container were fermented at 37 °C for 4 days. Soybeans fermented with *B. subtilis* KCTC 3135 and arbutin-producing *B. subtilis* strains were defined as positive control and experimental samples, respectively. Non-inoculated soybeans incubated under the fermentation conditions served as control. Soybean samples were taken every 24 h during the fermentation to measure pH, water activity, total mesophilic viable bacterial counts, arbutin content, and tyrosinase inhibitory activity.

### 2.5. Treatment of Arbutin Cultures and Soybean Samples for Arbutin Analysis and Tyrosinase Inhibitory Activity Assay

The arbutin content in arbutin cultures and soybean samples was determined according to the procedure by Park et al. [45] with slight modifications. The solvent for arbutin extraction (and for the HPLC mobile phase) was prepared with 10 mM monopotassium phosphate (Sigma) and acetonitrile (HPLC grade, SK Chemicals, Ulsan, Korea) at the ratio of 95:5 (*v*/*v*). For the arbutin extraction, 2 mL of the arbutin cultures (see Section 2.2) or 2 g of the soybean samples (see Section 2.4) were homogenized with 18 mL of the solvent prepared above using a vortex (Vortex-Genie, Scientific Industries, Bohemia, NY, USA; for the arbutin cultures) or a homogenizer (T10 basic Ultra-turrax, IKA, Staufen, Germany; for the soybean samples). The homogenates were sonicated with 400 W intensity at room temperature for 30 min using a JAC ultrasonic 4020 (Kodo Technical Research Co., Ltd., Daejeon, Korea), filtered through Whatman paper No. 1 (only for the homogenates from soybean samples; Whatman International Ltd., Maidson, UK), and subsequently filtered using a 0.45 μm-pore size syringe filter (Millipore).

Stock standard solutions of arbutin (>98%, Sigma) were prepared at a concentration of 10,000 mg/L in the solvent prepared above. Working standard solutions at concentrations of 0, 10, 25, 50, 100, and 250 mg/L were prepared by diluting the stock solution in the same solvent and filtered using a 0.45 μm-pore size syringe filter (Millipore).

All the filtrates from arbutin cultures, soybean samples, and standard solutions were stored at −70 °C until use. Within a week of storage, the filtrates were thawed in ice and directly used for arbutin analysis by HPLC (see Section 2.6). The filtrates were also used for the tyrosinase inhibitory activity assay (see Section 2.7).

### 2.6. Chromatographic Separation

Chromatographic separation of arbutin in the filtrates prepared above was conducted according to the method developed by Park et al. [45]. An HPLC unit (YL 9100, Young Lin Instrument, Co., Anyang, Korea) equipped with a UV–Vis detector (YL 9120, Young Lin) and Autochro-3000 software (Young Lin) was employed. A Nova-Pak C_18_ 5 µm column (250 mm × 4.6 mm, Waters, Milford, MA, USA) held at 25 °C was used for chromatographic separation. The mobile phase was adjusted to a flow rate of 1 mL/min. A 10 µL aliquot of each filtrate was injected and monitored at 260 nm for 15 min.

### 2.7. Tyrosinase Inhibitory Activity Assay

The tyrosinase inhibitory activity assay was carried out according to the method described by Piao, Baek, Park and Park [46] with minor modifications. Briefly, 40 μL of the filtrates prepared from each soybean sample or working standard solution (see Section 2.5) were added to a reaction mixture containing 40 μL of 0.01% (*w*/*v*) of L-tyrosine (in 0.067 M potassium phosphate buffer; pH 6.8), 80 μL of potassium phosphate buffer, and 40 μL of tyrosinase from mushroom (60 units/mL in potassium phosphate buffer; Sigma). As a blank, 40 μL of the solvent (described in Section 2.5) were added to the reaction mixture. The mixture was incubated at 37 °C for 30 min, and the optical density was measured at 475 nm using a spectrophotometer (Lambda 35, PerkinElmer Ltd., Waltham, MA, USA). Tyrosinase inhibitory activity was calculated as follows: Inhibition (%) = [1 − (A_sample_/A_blank_) × 100], where A_blank_ is the absorbance of the mixture with the solvent (blank) and A_sample_ is the absorbance of the mixture with the filtrate.

To estimate the tyrosinase inhibitory activity of arbutin in all soybean samples, a standard curve was used. The standard curve was generated using the filtrates prepared from the working standard solutions of arbutin at concentrations ranging from 0 to 250 mg/L. Arbutin content in the soybean samples was converted to tyrosinase inhibitory activity by using the standard curve plotting the concentration of arbutin versus tyrosinase inhibitory activity of the arbutin. The tyrosinase inhibitory activity of other substances was derived by the subtraction of that of arbutin from the total activity of soybean samples.

### 2.8. Physicochemical and Microbial Analyses

The physicochemical properties of soybean samples were measured as described below. Samples weighing 2 g using an analytical balance (Ohaus Adventurer™, Ohaus Corporation, Parsippany, NJ, USA) were homogenized with 18 mL of distilled water using a homogenizer. The pH of the homogenates was measured using a pH meter (Orion 3-star pH Benchtop Thermo Scientific, Waltham, MA, USA). The water activity of the samples was measured using an electric hygrometer (AquaLab Pre; Meter Group, Inc., Pullman, WA, USA).

The enumeration of total mesophilic viable bacteria in soybean samples was conducted using PCA as follows. Samples weighing 5 g were homogenized with 45 mL of sterile 0.1% peptone saline in a sterile plastic bag using a stomacher. The homogenates were 10-fold serially diluted with sterile 0.1% peptone saline up to 10^−6^, and 100 μL of each dilution was spread on PCA in duplicate. After incubation at 37 °C for 24 h, the bacterial concentrations of the soybean samples were calculated by counting the colonies on the plates with 10–300 colonies [39] and adjusting for the dilution.

### 2.9. Statistical Analyses

All measurements were performed in triplicates, while fermentation experiments were conducted in duplicates. Data were presented as means and standard deviations of duplicates or triplicates. Statistical analyses were performed with Minitab statistical software, version 17 (Minitab Inc., State College, PA, USA). The significance of differences was determined by one-way analysis of variance (ANOVA) with Fisher’s pairwise comparison, and differences with probability (*p*) value of <0.05 were considered statistically significant.

## 3. Results and Discussion

### 3.1. Arbutin Production by Bacillus Strains Isolated from Soybean-Based Products

In this study, *Bacillus* strains with arbutin production capability were used as a criterion for the selection of fermenting bacteria to ferment soybeans as (i) *Bacillus* spp. are generally involved in the fermentation of soybean-based products [29,30,47] and (ii) several species of the genus *Bacillus* have been reported to produce arbutin, an effective tyrosinase inhibitor as well as an outstanding antioxidant [19,20,41]. A total of 691 strains of *Bacillus* spp. were isolated from representative soybean-based products, including tofu, *Cheonggukjang*, *Gochujang*, *Chunjang*, *Doubanjang*, *Natto*, and *Miso*, and the arbutin production of each strain was measured by HPLC analysis. In conjunction, reference strains, including *B. subtilis* KCTC 3135, *B. licheniformis* KCTC 1918, *B. coagulans* KCTC 3625, and *B. pumilus* KCTC 3855, were also used as such species are dominant in soybean-based products [29,30,47].

**Figure 1:**
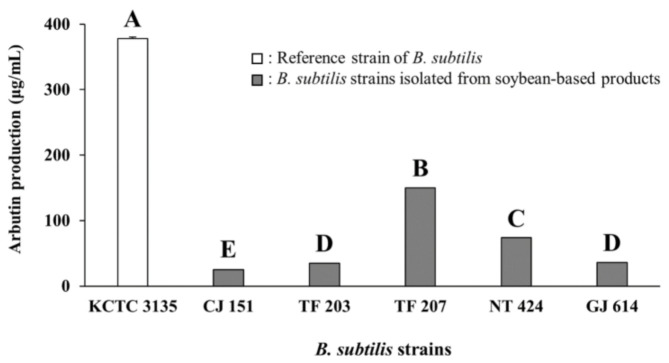
Arbutin production by various *B. subtilis* strains in assay media. KCTC 3135: Reference strain of *B. subtilis*, CJ 151: *B. subtilis* strain isolated from *Chunjang*, TF 203: *B. subtilis* strain isolated from tofu, TF 207: *B. subtilis* strain isolated from tofu, NT 424: *B. subtilis* strain isolated from *Natto*, GJ 614: *B. subtilis* strain isolated from *Gochujang.* Values of bars with different letters (A–E) are significantly different (*p* < 0.05). Error bars indicate standard deviations determined from triplicate experiments.

As presented in Figure 1 and Figure S1, *B. subtilis* KCTC 3135 (a reference strain) showed the highest production of arbutin (377.80 ± 7.08 μg/mL, mean ± standard deviation) among all tested strains, including reference and isolated strains. Of the *Bacillus* isolates, CJ 151 (isolated from *Chunjang*), TF 203, TF 207 (isolated from tofu), NT 424 (isolated from *Natto*), and GJ 614 (isolated from *Gochujang*) strains produced significantly higher levels of arbutin (25.24–150.05 μg/mL, the range from minimum to maximum). Moreover, the five *Bacillus* isolates also showed similar or higher arbutin production compared to reference strains of other species (14.04–38.72 μg/mL, Figure S1) except for *B. subtilis* KCTC 3135. The five isolates were all identified as *B. subtilis* based on 16S rRNA gene sequence analyses and selected for use in subsequent in situ fermentation experiments.

Some *Bacillus* spp. such as *B. subtilis*, *B. licheniformis*, *B. coagulans*, and *B. pumilus* which are indigenous to soybean-based products have been used for improving the functional properties of fermented soybean foods [29,30,47]. *B. subtilis* strains have also been considered prolific arbutin producers [19,41]. Particularly, Liu et al. [41] reported that while high levels of arbutin (100–500 mg/kg) were produced by *B. subtilis* reference strains, in vitro arbutin production was not observed with other reference strains of *B. licheniformis*, *B. pumilus*, and *B. amyloliquefaciens*. Similarly, in the present study, *B. subtilis* KCTC 3135 as well as CJ 151, TF 203, TF 207, NT 424, and GJ 614 strains identified as *B. subtilis* produced significantly higher levels of arbutin in assay media than the reference strains belonging to *B. licheniformis*, *B. coagulans*, and *B. pumilus*. Although the five *B. subtilis* strains were isolated from similar sources (i.e., soybean-based products), they had different arbutin production capabilities, showing a large variation with a standard deviation of 53.99 μg/mL. Based on previous and present studies, the arbutin production capability of *B. subtilis* strains is likely not only species-dependent but also strain-dependent [41]. Consequently, the present results suggest that arbutin-producing *B. subtilis* strains isolated from soybean-based products may have the potential to enhance the tyrosinase inhibitory activity (and antioxidative activity as well) of fermented soybean foods when used as fermenting bacteria. Furthermore, the fermented soybean foods prepared with these strains may be of interest to many researchers developing natural sources of cosmeceuticals and nutricosmetics for skin lightening and functional foods for improving health.

### 3.2. Changes in Physicochemical and Microbial Properties during Soybean Fermentation with Arbutin-Producing B. subtilis Strains

To examine the effects of the four arbutin-producing *B. subtilis* strains (CJ 151, TF 207, NT 424, and GJ 614; TF 203 was also tested but not described hereafter because only one strain with a stronger effect was selected from each soybean-based food source) on the physicochemical and microbial properties (Section 3.2), arbutin content (see Section 3.3), and tyrosinase inhibitory activity (see Section 3.4) of soybeans fermented with the respective strains, soybean fermentation experiments were performed using a *Cheonggukjang* model.

**Figure 2:**
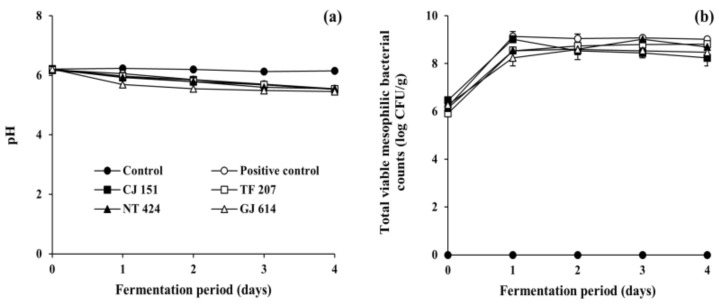
Changes in (**a**) pH and (**b**) total viable mesophilic bacterial counts of soybeans fermented with arbutin-producing *B. subtilis* strains. ●: control without an inoculum, ○: positive control inoculated with *B. subtilis* KCTC 3135, ■: experimental sample inoculated with *B. subtilis* CJ 151, □: experimental sample inoculated with *B. subtilis* TF 207, ▲: experimental sample inoculated with *B. subtilis* NT 424, △: experimental sample inoculated with *B. subtilis* GJ 614. Error bars indicate the minimum and maximum values of duplicate experiments.

As shown in Figure 2a, the initial pH of all inoculated soybean samples (positive control and all experimental samples) and non-inoculated soybean samples (control) ranged from 6.17 to 6.22. The pH of the control remained constant during the incubation period (corresponding to the fermentation period), while those of all the inoculated soybean samples steadily decreased to ranges of 5.45 to 5.85 as fermentation progressed, which is in agreement with previous studies on *Cheonggukjang* fermentation [43,44]. The reduction in pH may be associated with the growth of total mesophilic viable bacteria during the fermentation of soybeans (Figure 2b). The bacterial counts of all soybean samples inoculated with *B. subtilis* strains (reference and isolated strains) dramatically increased from the initial 6 log CFU/g to over 9 log CFU/g on day 1 of fermentation and stayed constant thereafter, which is in agreement with previous studies on *Cheonggukjang* [43,44]. Mesophilic viable bacteria were not detected in the control throughout the incubation period. In addition, the water activity of all soybean samples ranged from 0.988 to 0.992 throughout the experimental duration of fermentation or incubation. Altogether, the results of physicochemical and microbial measurements indicate that all the soybean samples inoculated with any of tested *B. subtilis* strains were properly fermented.

### 3.3. Changes in Arbutin Content during Soybean Fermentation with Arbutin-Producing B. subtilis Strains

**Figure 3:**
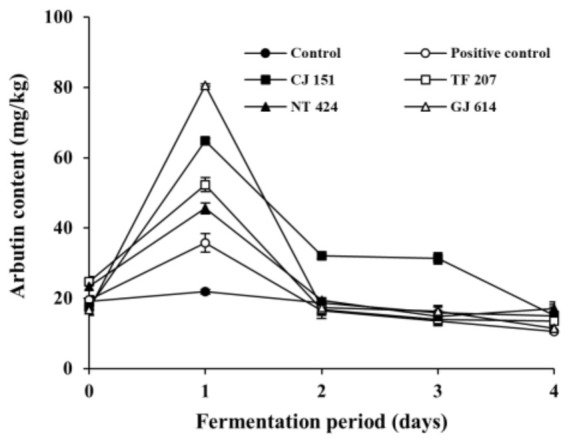
Changes in arbutin content in soybeans fermented with arbutin-producing *B. subtilis* strains. ●: control without an inoculum, ○: positive control inoculated with *B. subtilis* KCTC 3135, ■: experimental sample inoculated with *B. subtilis* CJ 151, □: experimental sample inoculated with *B. subtilis* TF 207, ▲: experimental sample inoculated with *B. subtilis* NT 424, △: experimental sample inoculated with *B. subtilis* GJ 614. Error bars indicate the minimum and maximum values of duplicate experiments.

To examine the arbutin production by arbutin-producing *B. subtilis* strains in fermented soybeans, arbutin content was analyzed during the fermentation of soybeans (Figure 3). The initial arbutin content in all inoculated soybean samples (positive control and all experimental samples) and non-inoculated soybean samples (control) was detected at approximately 20 mg/kg. Arbutin content in the control slightly decreased throughout the incubation period, while that in all the inoculated soybean samples dramatically increased on day 1 of fermentation and gradually decreased thereafter. Interestingly, arbutin content in the soybean samples inoculated with *B. subtilis* GJ 614 or *B. subtilis* CJ 151 was detected highest (80.58 ± 0.36 mg/kg) and second-highest (64.80 ± 0.68 mg/kg) on day 1 of the fermentation, respectively, compared to the other inoculated soybean samples. In contrast, the positive control inoculated with *B. subtilis* KCTC 3135 contained the lowest level of arbutin (35.81 ± 2.64 mg/kg) among all the inoculated soybean samples on the same day. It is noteworthy that both *B. subtilis* GJ 614 and *B. subtilis* CJ 151 produced relatively low levels of arbutin in assay media compared to the other selected *B. subtilis* strains, while *B. subtilis* KCTC 3135 showed the highest arbutin production in the same medium (see Figure 1). It is unclear why the difference in arbutin production by the *B. subtilis* strains between in vitro and in situ experiments occurred, but it is likely due to the different sources from which the strains were isolated. Indeed, *B. subtilis* CJ 151 and *B. subtilis* GJ 614 were isolated from *Chunjang* and *Gochujang*, respectively, whereas *B. subtilis* TF 207 and *B. subtilis* NT 424 were isolated from tofu and *Natto*, respectively. Although the four *B. subtilis* strains were isolated from similar sources (i.e., soybean-based products, as aforementioned in the Section 3.1), the former strains, but not the latter, were isolated from Korean fermented soybean foods similar to *Cheonggukjang* used as a model in this study. Contrastingly, *B. subtilis* KCTC 3135 was isolated from blood [48]. Thus, it seems that the two strains (*B. subtilis* CJ 151 and *B. subtilis* GJ 614) might be more adapted to the *Cheonggukjang* model system used in this study and thereby produced a larger amount of arbutin, compared to the other *B. subtilis* strains tested.

Hydroquinone has been known to be the primary precursor of arbutin [12]. Although studies related to hydroquinone content in soybeans have not been found in literature, arbutin production in fermented soybeans was observed in the present study. Therefore, it seems likely that for arbutin production, precursors other than hydroquinone are present in soybeans. A recent review described the formation of hydroquinone from polyphenols via the phenol moiety oxidation pathways [49]. As polyphenols are abundantly present in soybeans [29,30], the phenolic compounds might be utilized as precursors of arbutin during the fermentation of soybeans.

**Figure 4:**
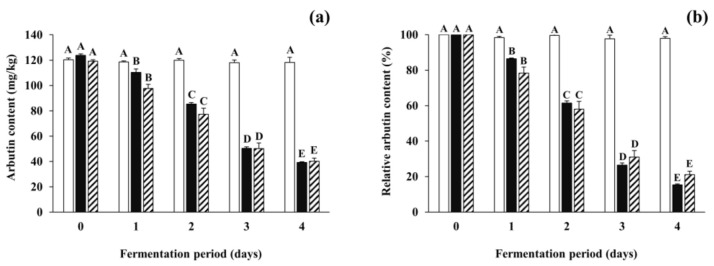
The degradation of arbutin spiked in soybeans fermented with arbutin-producing *B. subtilis* strains. (**a**) The total arbutin content (the total amount of arbutin naturally present in soybeans in addition to spiked arbutin); (**b**) the relative content of spiked arbutin. Arbutin was added in respective soybean samples at a concentration of 100 mg/kg. The relative content of arbutin was represented as the percentage of spiked arbutin content remaining in fermented or non-inoculated soybeans. □: control without an inoculum, ■: positive control inoculated with *B. subtilis* KCTC 3135, ▨: experimental samples (mean values). The mean values of striped bars were calculated from different experimental samples inoculated with each of the four *B. subtilis* strains (*B. subtilis* CJ 151, TF 207, NT 424, and GJ 614). Values of bars in the same color with different letters (A–E) are significantly different (*p* < 0.05). Error bars indicate standard deviations determined from duplicate experiments (control and positive control) or different experimental samples (experimental samples).

Arbutin content in all inoculated soybean samples gradually decreased from 35.81–80.58 mg/kg to 10.55–20.65 mg/kg after the first day of fermentation. To pursue the reasons for the reduction of arbutin, further fermentation experiments were carried out using the *B. subtilis*-inoculated soybean samples (and non-inoculated samples) spiked with arbutin at a concentration of 100 mg/kg, and arbutin content and total mesophilic viable bacteria counts were analyzed during the fermentation period. Although the content of spiked arbutin in the control slightly (but insignificantly) decreased during the incubation period, those in all the inoculated soybean samples significantly decreased by 77.13–84.64% during the fermentation period (Figure 4). Changes in total mesophilic viable bacterial counts of all the spiked samples were similar to those of non-spiked samples described in Section 3.2 (data not shown). The results indicate that the arbutin-producing *B. subtilis* strains not only produce arbutin in the early period of fermentation, but also degrade it throughout the fermentation period. A previous report described that the enzyme β-glucosidase of *B. subtilis* could degrade some phenolic glycosides such as arbutin and salicin into, for example, glucose, which in turn might be used as one of the carbon sources [50]. However, the dynamic nature of microbial mechanisms regulating both degradation and production of arbutin, particularly in fermented soybean foods such as *Cheonggukjang*, is still unclear. Based on the current findings, it would be interesting for further studies to optimize fermentation conditions that maximize arbutin production and minimize its degradation, which can then be extended to applications including the enhancement of the skin-lightening effect and anti-neurodegenerative activity of fermented soybeans with arbutin-producing *B. subtilis* strains.

### 3.4. Changes in Tyrosinase Inhibitory Activity during Soybean Fermentation with Arbutin-Producing B. subtilis Strains

**Figure 5:**
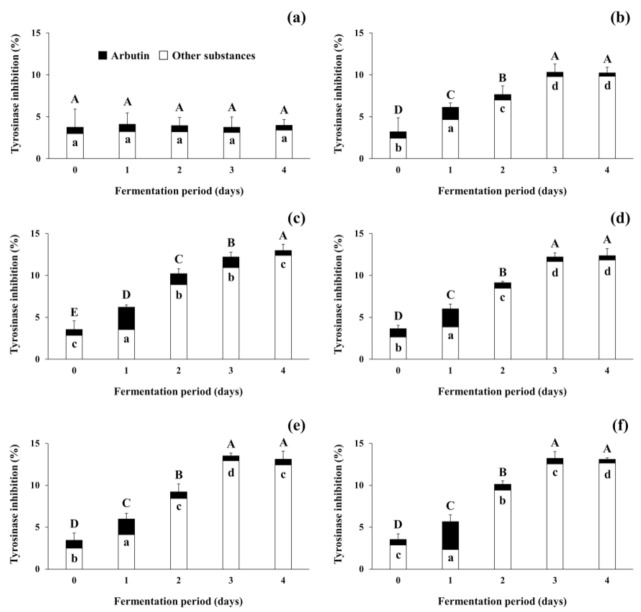
Changes in tyrosinase inhibitory activity of soybeans fermented with arbutin-producing *B. subtilis* strains. (**a**) The control without an inoculum; (**b**) the positive control inoculated with *B. subtilis* KCTC 3135; (**c**) the experimental sample inoculated with *B. subtilis* CJ 151; (**d**) the experimental sample inoculated with *B. subtilis* TF 207; (**e**) the experimental sample inoculated with *B. subtilis* NT 424; (**f**) the experimental sample inoculated with *B. subtilis* GJ 614. Arbutin content in soybean samples was converted to tyrosinase inhibitory activity by referring to a standard curve, and the activity of other substances was derived by the subtraction of that of arbutin from the total activity of soybean samples. ⬒: total tyrosinase inhibitory activity of soybean samples, ■: tyrosinase inhibitory activity of arbutin in soybean samples, □: tyrosinase inhibitory activity of other substances in soybean samples. The mean values of the total tyrosinase inhibitory activity (full bars, capital letters, A–E) or the activity of arbutin (black bars, small letters, a–d) that are not followed by the same letter are significantly different (*p* < 0.05). Error bars indicate half range of duplicate experiments.

The present study focused on tyrosinase inhibitory activity of the fermented soybeans because numerous previous studies on increases in antioxidants in fermented soybeans and their antioxidative activity have been reported [37,38]. As shown in Figure 5, tyrosinase inhibitory activity was observed during the fermentation of soybeans. The tyrosinase inhibitory activity of the non-inoculated soybean samples (control) stayed constant throughout the incubation period. In contrast, the activity of the positive control increased from 3.22 ± 1.66% (standard deviation from duplicate runs) to 10.23 ± 0.67%, and all 4 experimental samples showed larger increases in inhibitory activity, from 3.56 ± 0.09% (standard deviation from different samples) up to 12.89 ± 0.36% during the fermentation period. Differently from the present study using filtrates from fermented soybeans, several previous studies [25,34,35] have observed the tyrosinase inhibitory activity of lyophilized extracts from fermented soybeans. As lyophilization concentrates the substances in the samples, the treatment may be able to concentrate the inhibitory substances in the soybean samples tested in this study. However, changes in the activity and stability of soybeans fermented with arbutin-producing *B. subtilis* strains when exposed to such lyophilization conditions need to be further studied in the future. This is due to the possibility that such treatment may become an essential process in the use of fermented soybeans as a natural source of cosmeceuticals and nutricosmetics for skin lightening and functional foods for improving health. Regardless of the difference in sample preparation methods, the tyrosinase inhibitory activity of all fermented soybean samples in both previous and present studies increased as fermentation progressed.

In the meantime, to predict the tyrosinase inhibitory activity that was attributed to arbutin present in the control and all inoculated soybean samples, arbutin content was converted to inhibitory activity by referring to the standard curve (see Section 2.7; data not shown). The activity derived from arbutin was marked by black bars, whereas that from other substances was represented by white bars in Figure 5. The initial tyrosinase inhibitory activity attributed to arbutin content accounted for approximately 20% of total activity in all soybean samples. The remaining 80% activity appeared to be due to other substances besides arbutin. In the control, insignificant changes in the inhibitory activity were observed during the incubation period. However, in all the inoculated soybean samples, the tyrosinase inhibitory activity derived from arbutin increased to 23.93–58.74% of the total activity on day 1 of fermentation. The contribution of arbutin to the total activity decreased thereafter, to a minimum of 3.45–5.24% on day 4 of fermentation. The results indicate that arbutin produced by *B. subtilis* strains enhanced the tyrosinase inhibitory activity of fermented soybeans in the early period of fermentation, while other substances present in soybeans and produced by the *B. subtilis* strains contributed to the activity in the later period. Several compounds such as peptides in soybeans have been considered as tyrosinase inhibitors [51] and antioxidants [52]. Choi et al. [34] also observed that both tyrosinase inhibitory activity and antioxidative activity were moderately correlated to total phenolic content in *Cheonggukjang*. A previous study by Pyo and Jin [35] reported that the content of coenzyme Q10 and some soy isoflavones, including daidzein and genistein, in *Doenjang* significantly increased as fermentation progressed and contributed to both tyrosinase inhibitory activity and antioxidative activity. The results of the present study, taken together with previous studies, suggest that the fermentation of soybeans by arbutin-producing *B. subtilis* strains may enhance the tyrosinase inhibitory activity (and antioxidative activity as well) via the production of arbutin and other substances in fermented soybeans. After all, the optimization of fermentation conditions and selection of prolific arbutin producers to properly ferment soybeans will enable fermented soybeans to be used as beneficial materials in the medicine and cosmetics industry as well as the food industry.

## 4. Conclusions

In this study, prolific arbutin-producing *B*. *subtilis* isolates were selected and used for the fermentation of soybeans to enhance tyrosinase inhibitory activity (eventually resulting in skin-lightening effect and anti-neurodegenerative activity). Arbutin content and tyrosinase inhibitory activity thereof in all soybean samples fermented with the arbutin-producing *B*. *subtilis* strains significantly increased in the early period of fermentation and decreased thereafter, which, together with the other results of the present study, indicated that the *B*. *subtilis* strains were usable to enhance the tyrosinase inhibitory activity of the fermented soybeans, and also capable of degrading arbutin throughout the fermentation period. In the meantime, the total tyrosinase inhibitory activity increased as fermentation progressed, which implied that other substances (besides arbutin) present in soybeans and produced by the *B. subtilis* strains contributed to tyrosinase inhibition. Therefore, the current study suggests that optimization of fermentation conditions to maximize microbial arbutin production and minimize its degradation is necessary to further enhance the tyrosinase inhibitory activity (and antioxidative activity if required) of soybeans fermented with the selected *B. subtilis* strains. In addition, as the arbutin-producing *B. subtilis* strains originated from soybean-based products, soybeans fermented with the strains may be favorable in the production of beneficial materials to be used in the medical and cosmetics industries, as well as the food industry.

## Supplementary Materials

The following are available online at https://www.mdpi.com/2076-3921/9/12/1301/s1, Figure S1: Arbutin production by reference strains of *Bacillus* spp. in assay media. The black bar represents the mean value of arbutin production by five *B. subtilis* isolates (CJ 151, TF 203, TF 207, NT 424, and GJ 614) selected based on arbutin production capability. Values of bars with different letters are significantly different (*p* < 0.05). Error bars indicate standard deviations determined from triplicate experiments (each reference strain) or different *B. subtilis* isolates (isolated strains).
