# Supplementary material for: Tyrosinase Inhibitory Activity of Soybeans Fermented with Bacillus subtilis Capable of Producing a Phenolic Glycoside, Arbutin
Source: Antioxidants (Basel). 2020 Dec 18;9(12):1301. doi: 10.3390/antiox9121301 (PMC7766266; doi:10.3390/antiox9121301)
Supplement: Supplementary file 1 [file antioxidants-09-01301-s001.zip › 2020-antioxidants-arbutin fermented soybean-proofreading-supplementary material.docx]

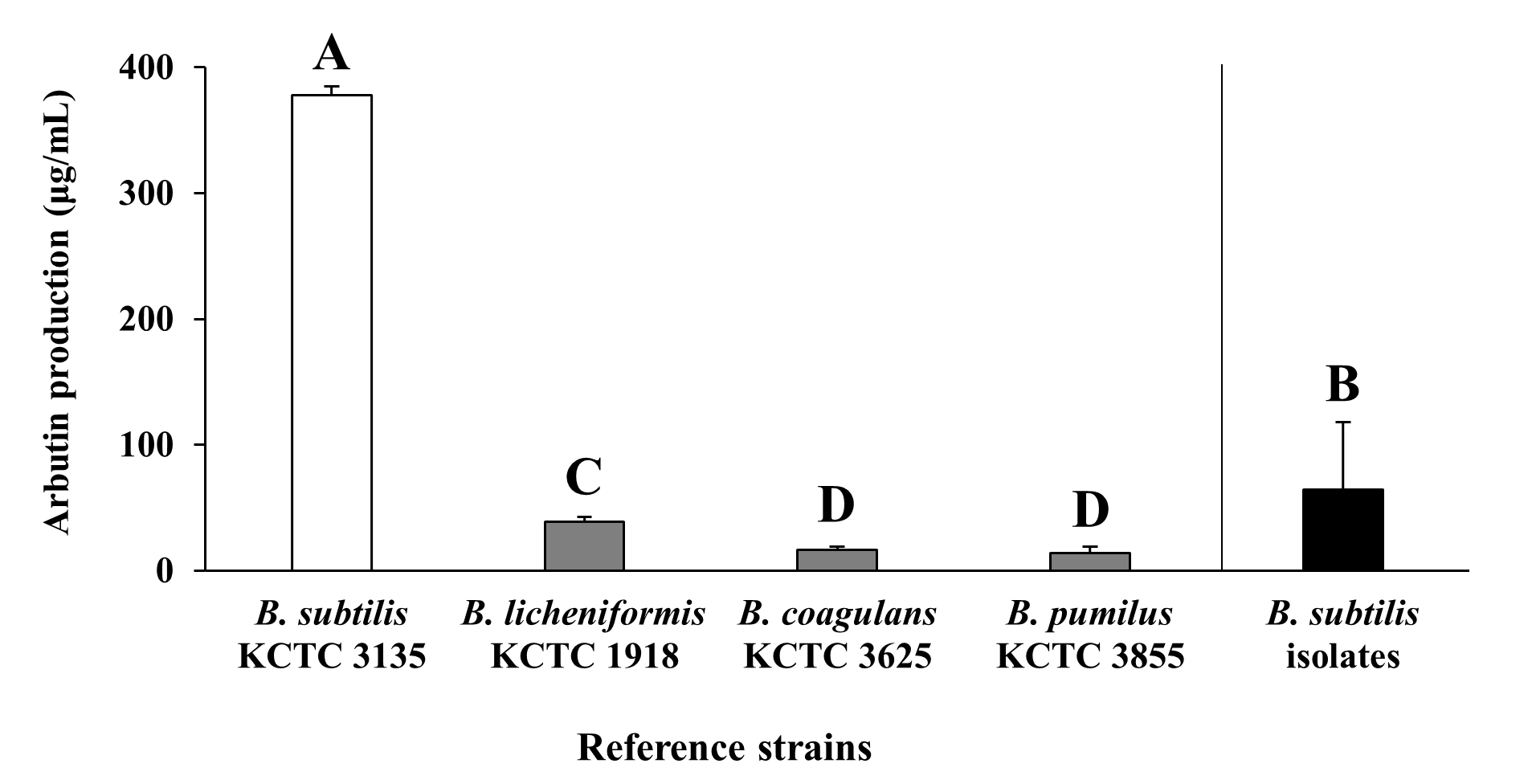


**Figure S1.** Arbutin production by reference strains of *Bacillus* spp. in assay media. The black bar represents the mean value of arbutin production by five *B. subtilis* isolates (CJ 151, TF 203, TF 207, NT 424, and GJ 614) selected based on arbutin production capability. Values of bars with different letters are significantly different (*p* < 0.05). Error bars indicate standard deviations determined from triplicate experiments (each reference strain) or different *B. subtilis* isolates (isolated strains).
